# Supplementary material for: Biochemical, Biophysical, and Structural Analysis of an Unusual DyP from the Extremophile Deinococcus radiodurans
Source: Molecules. 2024 Jan 11;29(2):358. doi: 10.3390/molecules29020358 (PMC10820274; doi:10.3390/molecules29020358)
Supplement: Supplementary file 1 [file molecules-29-00358-s001.zip › Supplementary.pdf]

Supplementary Figures

Biochemical, biophysical and structural analysis of an unusual DyP from the extremophile *Deinococcus radiodurans*

Kelly Frade[1], Célia M. Silveira[1], Bruno A. Salgueiro[1], Sónia Mendes[1], Lígia O. Martins[1], Carlos Frazão[1], Smilja Todorovic[1] and Elin Moe[1,*]

[1]Instituto de Tecnologia Química e Biológica António Xavier (ITQB-NOVA), Universidade Nova de Lisboa, Av. da Republica (EAN), 2780-157 Oeiras, Portugal.

[*]Corresponding author (elinmoe@itqb.unl.pt)

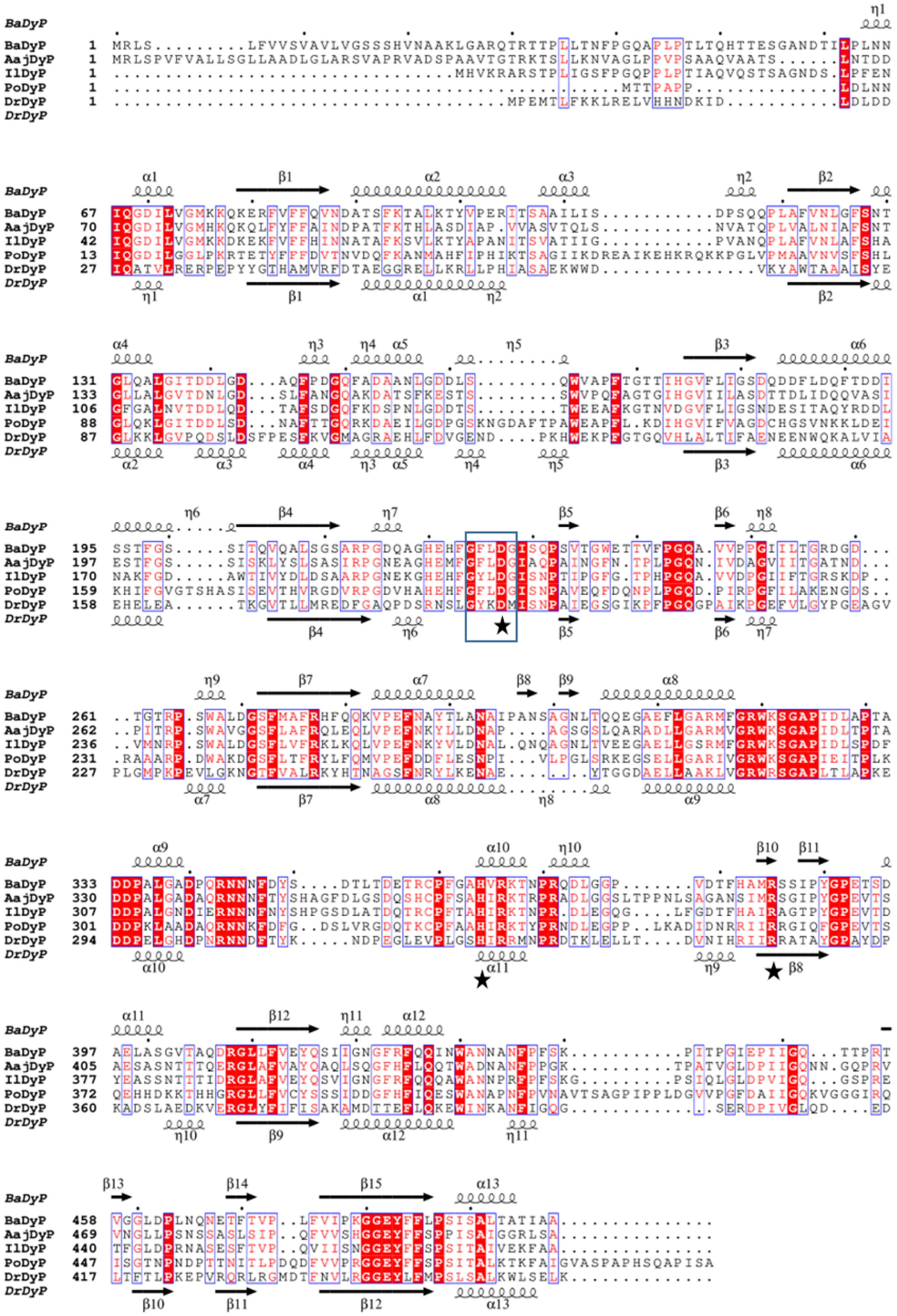

Figure S1. Sequence alignment of *Dr*DyP (bottom sequence) and four previously structurally determined family IV DyPs; *Bjerkandera adusta* DyP (*Ba*DyP, PDB ID: 2D3Q), *Auricularia auricula-judae* DyP (*Aaj*DyP, PDB ID: 4AU9), *Irpex lacteus* (*Il*DyP, PDB ID: 7D8M) and *Pleurotus ostreatus* DyP (*Po*DyP, PDB ID: 6FSK/L). The position of the conserved heme ligating histidine (His324), the catalytic Asp and Arg are indicated with black stars. The so-called GXXDG motif is indicated in a blue box.

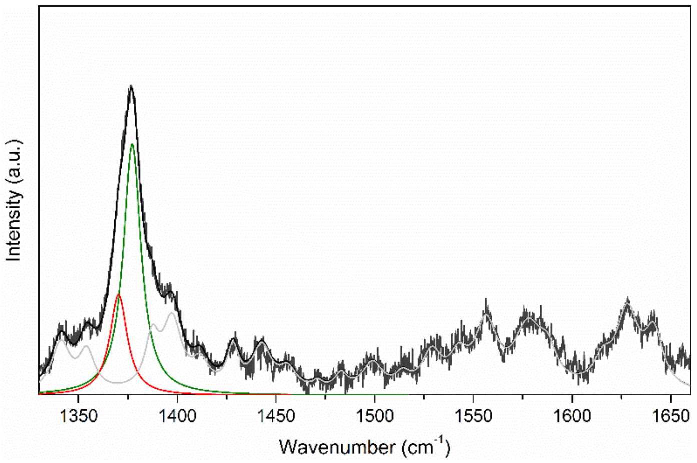

Figure S2. RR spectrum of DrDyP measured at pH 3. The component spectra represent the 6cLS and 6cHS populations in green and red, respectively. Due to the low spectral quality, only the ν_4 region was analyzed; the non-assigned bands are shown in light gray. The spectrum was acquired at room temperature with 413 nm laser excitation in citrate-phosphate buffer solution adjusted to pH 3.

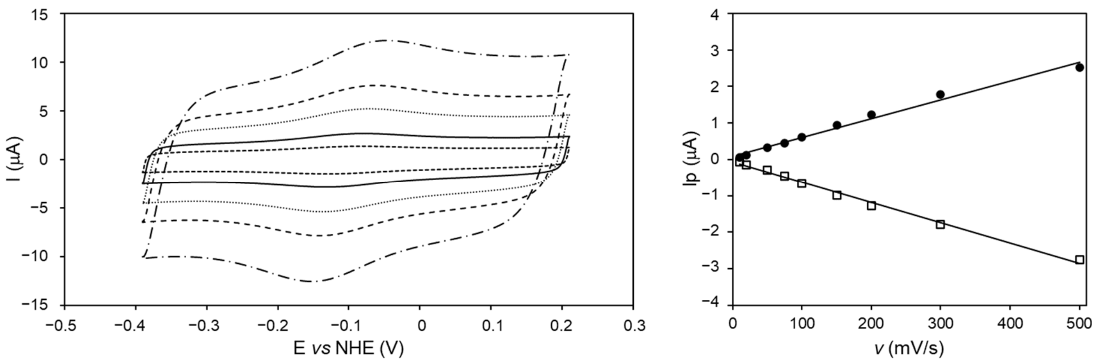

Figure S3. Electrochemical analysis. **Left panel**: Cyclic voltammograms of DrDyP adsorbed on pyrolytic graphite electrodes at scan rates (v) from 20 to 500 mV/s (inner to outer scans). **Right panel**: Peak current as a function of the scan rate. Cathodic and anodic peak currents are represented by the open squares and black circles, respectively. Measurements performed in 50 mM KCl in 40 mM BR buffer, pH 7.

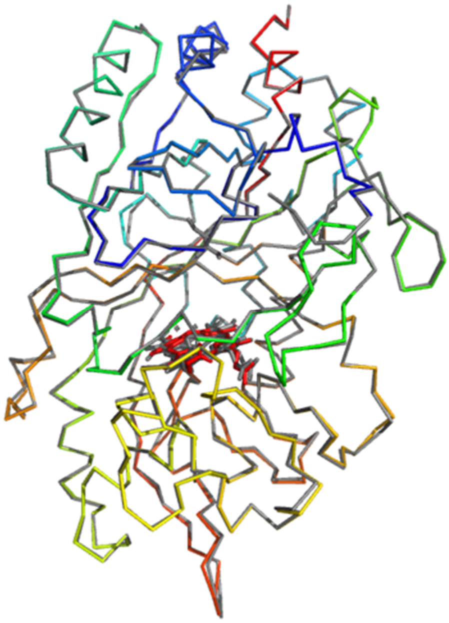

Figure S4. Structure superpositioning**.** Superpositioning of the backbone of *Dr*DyP and *Dr*DyPM190G. DrDyP is shown in rainbow colours, while the mutant is shown in grey.
